# Role of colon capsule Pillcam 2 in obscure gastrointestinal bleeding - case report

**Published:** 2013-09-25

**Authors:** RD Babiuc, M Purcarea, R Sadagurschi, L Negreanu, T Nastasescu

**Affiliations:** * 2nd Medical Clinic of Gastroenterology, University Emergency Hospital; ** 2nd Nephrology Department, “Carol Davila" Clinical Nephrology Hospital; *** 1st General Surgery Department, University Emergency Hospital

**Keywords:** videocapsule endoscopy, obscure gastrointestinal bleeding, colon cancer

## Abstract

**Introduction:** The diagnosis and management of gastrointestinal bleeding have always been a challenge to clinicians. In most patients, the source of bleeding is easily identified during conventional upper and/or lower gastrointestinal endoscopies. A significant progress in the evaluation of patients with obscure gastrointestinal bleeding was brought by the advent of capsule endoscopy. Since colonoscopy is not always technically feasible, colon VCE might be useful where the conventional procedure poses substantial risks to patients or it is refused by them. **Case-report:** We present the case of a 58-year-old patient, with severe anemia caused by bleeding from a gastrointestinal source. The patient was diabetic, hypertensive and with impaired heart function, aggravated by anemia. We used the Pillcam Colon 2 capsule to investigate the colon and we found 2 tumors in the cecum and transverse colon. **Conclusion:** Pillcam Colon 2 capsule turned out to be an additional patient-friendly method to complement colonoscopy for colon visualization and colorectal cancer screening.

## Introduction

The diagnosis and management of gastrointestinal bleeding have always been a challenge to clinicians. The condition is a common presentation of a wide array of lesions along the gastrointestinal tract [**1**]. In most patients, the source of bleeding is easily identified during conventional upper and/or lower gastrointestinal endoscopies. Since its introduction into practice in 2001, small bowel capsule endoscopy (SBCE) was used in the diagnosis of obscure bleeding and brought a significant progress to the evaluation of such patients [**2**]. An esophageal and a colon capsule have also been launched on the market and are under intensive clinical investigation [**3**]. A second-generation, improved CCE system (PillCam Colon 2) was developed to increase sensitivity for colorectal polyp detection compared with the first-generation system. A recent study using a second-generation colon capsule showed a higher sensitivity for detection of patients with significant colonic lesions (11) of almost 90%. Since colonoscopy is not always technically feasible, colon VCE might be useful first as a complement to incomplete colonoscopy, and secondly where conventional colonoscopy is either refused by patients or poses substantial risk to them [**3**].

## Case report

**Fig. 1:**
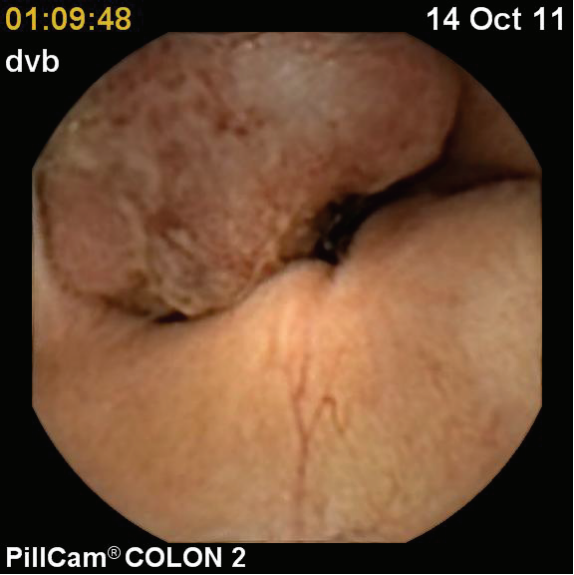
Cecal tumor

**Fig. 2:**
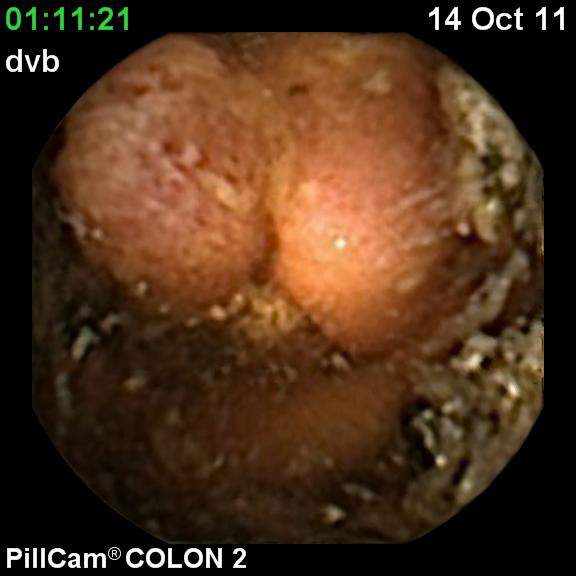
Second tumor transverse colon

**Fig. 3:**
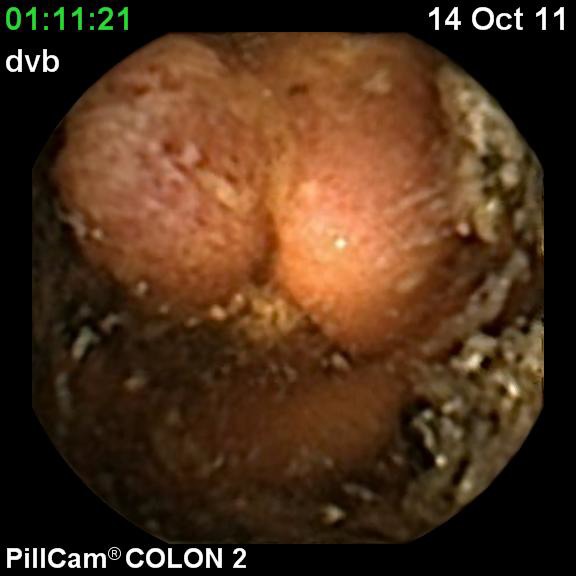
Second tumor, close view

We report the case of a 58-year-old woman diagnosed with multiple simultaneous colon cancer using the Pillcam Colon 2. The patient referred to our service for dyspnea, fatigue and chest pain with a progressive onset. From patient history, we should mention type II diabetes mellitus, atrial fibrillation and cardiac ischemic disease and family history of colon cancer (father). She received a low dose of aspirin (Aspenter 75mg). The patient denied melena or changes of bowel habits. On examination, the patient was noted to be pale, tachycardic (pulse = 104 bpm), orthopneic, with pulmonary rales, tender liver and periferal pitting edema; gynecologic evaluation was normal. Blood tests showed severe hypochromic-microcytic anemia (hemoglobin = 7,61g/dl; MCV=66,2fl; MCH=19,8pg), thrombocytosis (PLT= 500.000/mmc) with low level of serum Fe (19 microg/dl) and high Fe-binding capacity (495 mg/dl). Renal function tests revealed a minimally elevated BUN (51mg/dl). Other biochemical markers found some inflammatory activity (ESR = 50mm/h, CRP +), while tumoral markers were non-reactive. Upper endoscopy revealed an erosive pangastritis (probably due to aspirin ingestion) with no evidence of bleeding. This is why we decided to perform a colonoscopy, but because of the associated co-morbidities, the patient could not tolerate the examination. We used the Pillcam colon 2 system to investigate the entire digestive tract. The patient underwent a special preparation regimen with 6L of PEG (2L one day before capsule ingestion, 2L in the same day before ingestion and 2L after ingestion). This revealed one tumoral mass in the caecum (**Fig. 1**) and a second tumor in the transverse colon (Fig. 2,3). After blood transfusions (2 MER units) and improvement of cardiac function, the patient was transferred in a surgery unit and two days later, she underwent surgery. Because the preoperative CT scan found no metastatic lesions and lymph nodes, there was no need for chemotherapy. The follow-up at 3 and 6 months after surgery identified no early complications or tumor recurrence.

## Discussion

Diseases of the colon are major health problems in many parts of the world. Colonoscopy allows direct visualization of the colon and is currently the procedure of choice for the evaluation of colorectal disease and has a major role in colorectal cancer screening programs [4,5]. However, there are challenges associated with this procedure and barriers to its use (invasiveness, sedation, insufflation, perforation, and loss of working days). Many patients are reluctant to undergo this procedure for various reasons, including fear of discomfort and inconvenience as well as psychological inhibitions [**6**]. An important progress in the evaluation of patients with obscure gastrointestinal bleeding was made by with the introduction into practice of the capsule endoscopy. The new second-generation colon capsule endoscopy (Pillcam Colon 2) is a safe and effective method for the visualization of the colon and the detection of colonic lesions without the need for sedation, intubation, or air insufflations [**6**]. It is slightly longer than the previous generation with 11.6 X 31,5 mm in size. It has been designed to work for at least 10 hours and it has a variable frame rate (from 4 to 35 frames/second in order to correctly visualize the mucosa when accelerated peristalsis). The angle of view was increased to 172 degrees in both capsule lenses, thus covering almost 360 degrees of the colonic surface. A new smaller and more ergonomic data recorder with a liquid crystal display allowing real time viewing was developed. It permits a bidirectional communication with the capsule and also is friendlier and easier to use by the patient providing automatic visual and audio signals for procedure activities (boost administration). Sensitivity and specificity for the detection of colorectal polyps appear to be very good; Eliakim et al. found sensitivity in the detection of polyps in some patients ≥6mm and ≥10 of 89% and 88%, respectively. In the same study, the capsule specificities for the detection of polyps in patients ≥6mm and ≥10mm are 76% and 89%, respectively [**3**]. A major drawback of this procedure is its high cost, which makes it unavailable in common practice. However, in isolated cases like ours, the colon VCE seems to be useful and effective, enabling us to establish a diagnose and avoid further complications.

## Conclusions

Our report highlights the role of colon capsule endoscopy in the evaluation of the colon when colonoscopy has failed, has been rejected by the patient, or when anesthesia is contraindicated. It is thought to be an additional patient-friendly method to complement colonoscopy for colon visualization and colorectal cancer screening [**6**].
